# Effect of Prepregnancy Lymphocyte Active Immunotherapy on Unexplained Recurrent Miscarriage, Pregnancy Success Rate, and Maternal-Infant Outcome

**DOI:** 10.1155/2021/7878752

**Published:** 2021-10-14

**Authors:** Junxia Li, Yan Gu, Shaojing Zhang, Baohui Ju, Jianmei Wang

**Affiliations:** Department of Obstetrics and Gynecology, Second Hospital Affiliated to Tianjin Medical University, China

## Abstract

**Objective:**

To evaluate the effect of prepregnancy lymphocyte active immunotherapy on unexplained recurrent miscarriage, pregnancy success rate, and maternal-infant outcome.

**Methods:**

A total of 124 patients with recurrent miscarriage admitted to our hospital from January 2018 to December 2020 were selected as the research objects and divided into the experimental group and the control group according to the random number table method, with 62 patients in each group. The experimental group was treated with lymphocyte active immunotherapy, and the control group was given conventional treatment. The pregnancy success rate, estrogen indexes, hemorheology indexes, and psychological state of the two groups were compared.

**Results:**

The experimental group garnered a notably higher pregnancy success rate and a prominently lower miscarriage rate than the control group (*P* < 0.05). Better results of self-rating anxiety scale (SAS) and self-rating depression scale (SDS) were observed in the experimental group, as compared to the control group (*P* < 0.05). The experimental group yielded more desirable results in terms of treatment satisfaction, estrogen indexes, and hemorheology indexes in comparison with the control group (*P* < 0.05).

**Conclusion:**

The use of lymphocyte active immunotherapy for patients with unexplained recurrent miscarriage can significantly increase the pregnancy success rate, optimize the maternal-infant outcome, drive down the miscarriage rate, and ameliorate the patient's estrogen levels and hemorheology indicators, which is worthy of promotion and application in clinical practice.

## 1. Introduction

The spontaneous miscarriage that occurs twice or more in a row is called recurrent miscarriage, and that occurs three times or more in a row is habitual miscarriage. Only 50% of patients with recurrent miscarriage can be diagnosed with specific causes which mainly include unhealthy lifestyle, maternal endocrine abnormalities, maternal reproductive tract abnormalities, cervical insufficiency, chromosomal abnormalities, reproductive tract infections, and chromosomal abnormalities, while the cause of the other 50% of patients still remains not clear [1–3]. Relevant studies in recent years have shown that more than half of the causes of unexplained recurrent miscarriage, which has been witnessed an increasing trend recently, are closely related to immune dysfunction, that is, imbalance of pregnancy immune tolerance and abnormal maternal immune regulation. To date, consensus on the treatment of unexplained recurrent miscarriage has not yet been developed. Despite the safety and effectiveness of lymphocyte active immunotherapy are challenged at times, it is the most common therapy in clinical practice [4–6]. Therefore, this study is to assess the effect of prepregnancy lymphocyte active immunotherapy on unexplained recurrent miscarriage, pregnancy success rate, and maternal-infant outcome. The report is as follows:

## 2. Materials and Methods

### 2.1. General Information

A total of 124 patients with recurrent miscarriage admitted to our hospital from January 2018 to December 2020 were selected as the research objects and divided into the experimental group and the control group according to the random number table method, with 62 patients in each group.

### 2.2. Inclusion Criteria

(1) All patients met with the diagnostic criteria for unexplained recurrent miscarriage; (2) the chromosomal examinations of all couples were normal; (3) the endocrine function of the patients was normal; (4) the test results of anti-cardiolipin, anti-endometrial, anti-nuclear, and other antiautoantibodies of all patients were negative. (5) This study was approved by the hospital ethics committee. The patients and their families signed an informed consent form after being fully informed of the purpose and process of the study.

### 2.3. Exclusion Criteria

(1) The liver function test results of the couples were abnormal; (2) the patients had reproductive tract infections such as chlamydia, HIV, mycoplasma, and cytomegalovirus; (3) the semen examination of the patients' husband or a third party who provided lymphocytes was abnormal; (4) B-ultrasound examination showed that the patients' uterus had congenital malformations and intrauterine adhesions.

### 2.4. Methods

The control group received conventional treatment, given progesterone (National Medical Product Administration Approval Number H20041902; Zhejiang Xianju Pharmaceutical Co., Ltd.; 50 mg∗10 s∗2 plate), once/day, 100 mg/time, after meals, for 14 days. When the patient was diagnosed with pregnancy, intramuscular injection of human chorionic gonadotropin 2000 U was performed, 2 d/time. After a week of continuous injection, it was then changed to 2 times/week, and after two weeks of continuous injection, it was changed to 1 time/week. The treatment continued for 3 months.

The experimental group received lymphocyte active immunotherapy: (1) before active immunization, a blocking antibody test should be performed on empty stomach during pregnancy. 100 *μ*l of the patient's husband's peripheral anticoagulated whole blood was collected, 20 *μ*l of mouse anti-human CD3, CD4, and CD8 monoclonal antibodies was added, and then, 50 *μ*l of patient serum or normal male AB serum was added, incubated for 30 minutes; next, 3 ml of red blood cell lysis solution was added and washed twice with PBS; subsequently, a flow cytometry was used to analyze the ratio of CD3, CD4, and CD8 cells of spouse or third male lymphocytes with female serum and AB serum, and the blocking antibody efficiency was calculated according to the following formula: blocking efficiency = CD ratio with AB serum (%) − the proportion of female serum CD (%), the normal fertility group blocking efficiency is X-1.96 s, and those greater than this value is considered positive. If the test result was negative, the peripheral blood would be drawn again after 4 times of immunotherapy, and the blocking antibodies in the body would be checked in the same way [7]. (2) During the active immunization process, the couples were genotyped with sequence-specific primer PCR for HLA-II locus. The husband's lymphocytes are the first choice for active immunization. If the husband is not suitable as an immunogen donor (HBsAg positive), other healthy individuals should be selected. The consistency of the two sites indicated an involvement of a third party donor, and a blood type test for the both parties was performed. Subsequently, 20 ml of the donor's peripheral blood was drawn, and the lymphocytes were separated by Ficoll density gradient centrifugation and washed with sterile saline three times to remove platelets and form a lymphocyte suspension with a concentration of (2 ~ 4) × 107/mL. After disinfection of the skin on the inner forearm, 2 to 4 points were selected for subcutaneous injection (0.5 cm for the interval), with 0.2~0.3 mL each time at each point, 3 weeks/time, and a course of treatment contained 4 times of treatment. Contraception was indispensable during the treatment. After one course of treatment, if the test result of blocking antibodies was positive, the patient would be advised to conceive within three months; otherwise, lymphocyte immunotherapy would be continued till the test result turned into positive, and the patient then was permitted to get pregnant. The therapy lasted for 3 months [8, 9].

After the treatment, a six-month follow-up was carried out. The adverse reactions of the patients were recorded in detail and handled with timely and effective treatment.

### 2.5. Indicators Observation

The pregnancy success rate of the two groups was compared.

The “Patient Clinical Satisfaction Survey Questionnaire” prepared by the hospital was used to investigate the patients' satisfaction with treatment. The degree of satisfaction was divided into satisfied, basically satisfied, and dissatisfied. Total satisfaction = satisfied + basically satisfied.

Radioimmunoassay was used to measure the changes of estrogen indicators in the two groups of patients. The estrogen indicators contain progesterone, estradiol, and *β*-HCG.

Flow cytometer, viscometer, and electrophoresis instrument were used to detect hemorheology indexes of the two groups of patients. Hemorheology indexes include hematocrit (HCT), plasma viscosity (PV), erythrocyte sedimentation rate (ESR), and erythrocyte electrophoresis time (EET).

The SAS [10] was used to assess the psychological anxiety of the two groups of patients before and after intervention. The total score of the scale is 50 points. The higher the score, the more serious the patient's anxiety.

The SDS [11] was used to evaluate the depression of the two groups of patients before and after the intervention. The total score of the scale is 55 points. The higher the score, the more severe the depression of the patient.

### 2.6. Statistical Processing

The experimental data was statistically analyzed and processed by the SPSS21.0 software, and GraphPad Prism 7 (GraphPad Software, San Diego, USA) was used to plot graphics. The count data was represented by *n* (%), using *χ*^2^ test, and the measurement data was expressed as *x̅*±*s*, using *t*-test. A *P* value less than 0.05 was considered statistically significant.

## 3. Results

### 3.1. Comparison of Clinical Information between the Two Groups

The two groups did not show great disparity in terms of their general information such as mean age, mean height, mean pregnancy times, education level, and place of residence (*P* > 0.05) (see Table 1).

### 3.2. Comparison of Pregnancy Success Rate between the Two Groups after Treatment

Table 2 demonstrates a higher pregnancy success rate and a lower miscarriage rate of the experimental group than that of the control group (*P* < 0.05).

### 3.3. Comparison of SAS Scores between the Two Groups before and after Treatment

**Figure 1:**
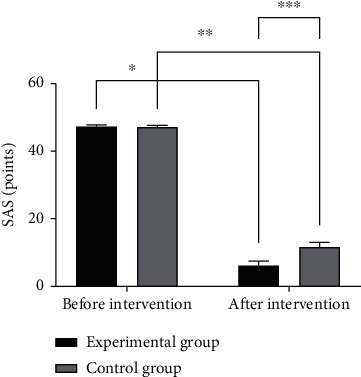
Comparison of SAS scores between the two groups before and after treatment (*x̅*±*s*). Note: the abscissa represents the experimental group and control group after treatment, and the ordinate represents the SAS scores, points. The SAS scores of the experimental group before and after treatment are 47.33 ± 0.51 points and 6.21 ± 1.33 points, respectively. The SAS scores of the control group before and after treatment are 47.17 ± 0.48 points and 11.66 ± 1.43 points, respectively; ∗ indicates that the SAS scores of patients in the experimental group before and after treatment are significantly different (*t* = 227.304, *P* ≤ 0.01); ∗∗ indicates that the SAS scores of patients in the experimental group before and after treatment are significantly different (*t* = 185.365, *P* ≤ 0.01); ∗∗∗ indicates that the SAS scores of the two groups of patients after treatment are significantly different (*t* = 21.974, *P* ≤ 0.01).

The experimental group yielded a superior outcome of SAS scores when compared with the control group (*P* < 0.05) (see Figure 1).

### 3.4. Comparison of SDS Scores between the Two Groups before and after Treatment

**Figure 2:**
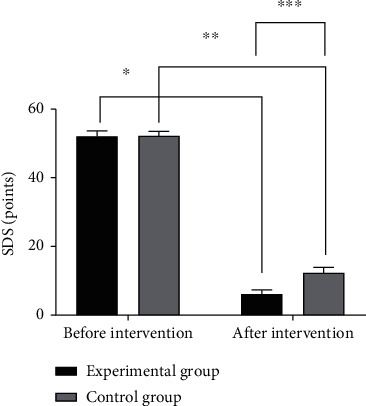
Comparison of SDS scores between the two groups (*x̅*±*s*). Note: the abscissa represents the experimental group and control group after treatment, and the ordinate represents the SDS scores, points. The SDS scores of the experimental group before and after treatment are 52.13 ± 1.6 points and 6.18 ± 1.2 points, respectively. The SDS scores of the control group before and after treatment are 52.21 ± 1.3 points and 12.33 ± 1.6 points, respectively; ∗ indicates that the SDS scores of patients in the experimental group before and after treatment are significantly different (*t* = 180.905, *P* ≤ 0.01); ∗∗ indicates that the SDS scores of patients in the experimental group before and after treatment are significantly different (*t* = 152.320, *P* ≤ 0.01); ∗∗∗ indicates that the SDS scores of patients in the experimental group before and after treatment are significantly different (*t* = 24.213, *P* ≤ 0.01).

The SDS scores of the experimental group after intervention were significantly better than that of the control group (*P* < 0.05), as shown in Figure 2.

### 3.5. Comparison of Treatment Satisfaction between the Two Groups

**Figure 3:**
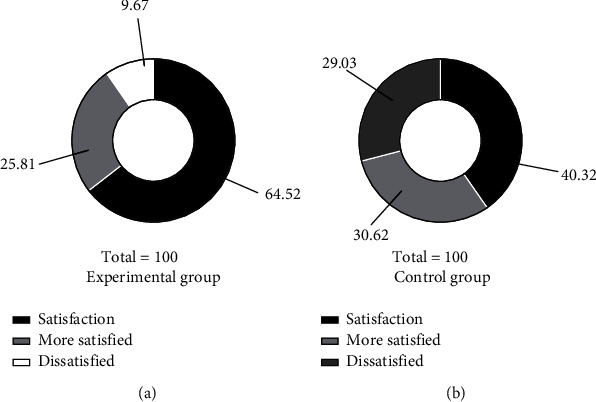
Comparison of satisfaction between the two groups (*n* (%)). Note: (a) the treatment effect in the experimental group; (b) the treatment effect in the control group. The satisfied rate of the experimental group is 64.52% (40/62), the basically satisfied rate is 25.81% (16/62), the dissatisfied rate is 9.67% (6/62), and the overall satisfaction rate is 90.32% (56/62). The satisfied rate of the control group is 40.32% (25/62), the basically satisfied rate is 30.65% (19/62), the dissatisfied rate is 29.03% (18/62), and the overall satisfaction rate is 75.81% (47/62). There is significant difference between the two groups of patients after treatment (*χ*^2^ = 6.719, *P* = 0.010).

Patients in the experimental group were more satisfied with the treatment than those in the control group (*P* < 0.05), as shown in Figure 3.

### 3.6. Comparison of Estrogen Indicators between the Two Groups of Patients

Strong evidence of higher estrogen indicators of the experimental group by contrast to the control group was found (*P* < 0.05) (see Table 3).

### 3.7. Comparison of Hemorheology Indexes between the Two Groups of Patients

Table 4 presents a significant difference in hemorheology indicators between the experimental group and the control group after treatment (*P* < 0.05).

## 4. Discussion

At present, the current childbearing age is generally delayed with the change of people's life concept, and a gradual rise in the incidence of recurrent miscarriage has been witnessed year by year. Recurrent miscarriage is considered as a refractory disease in consequence of its rather complicated causes in clinic, among which the miscarriage triggered by immune factors accounts for more than half [12–15]. In modern immunology, it is believed that the genetic antigens from the father during the embryo formation process are treated as foreign invaders, which triggers rejection reactions in the mother; hence, miscarriage indicates the failure of semihomogenous transplantation. The body of normal pregnant women will produce blocking antibodies on their own to fight against natural rejection, thus ensuring the growth and development of the embryo. However, the deficiency of blocking antibodies in the mother will consequently lead to the attack from the mother's natural rejection on the embryo which eventually results in an abnormal gestation [16–20]. Such shortage of blocking antibodies can be remedied by the implantation of lymphocyte active immunotherapy into the patient's body through allogeneic lymphocytes to stimulate the patient's immune system. When the patient is pregnant again, the blocking antibodies that have been produced can be recognized by the homologous antibodies to ensure the protection of the embryo growth. Early recurrent miscarriage may also be related to the patient's low response to embryos and semihomogenous antigens, embryo rejection, and inability to produce suitable blocking antibodies. The deficiency of blocking antibodies is one of the main reasons behind recurrent miscarriage and the termination of embryo development. Studies have shown that more than 85% of terminated development of embryos and recurrent miscarriage are linked to the shortage of blocking antibodies in patients [21–24]. The use of lymphocyte immunotherapy can substantially elevate the transformation rate of blocking antibodies in patients from negative to positive. The results of this study showed prominently higher levels of estrogen indicators of the experimental group than those of the control group (*P* < 0.05), which was consistent with the research results of Salimi et al. [25] who pointed out that “after treatment, the indexes of estradiol, progesterone, and *β*-HCG in the experimental group were 138.81 ± 20.43, 32.33 ± 5.51, and 24.12 ± 4.35, respectively, and in the control group were 113.31 ± 46.72, 32.29 ± 5.54, and 24.09 ± 4.37, respectively. The estrogen indicators of the experimental group were significantly higher than those of the control group (*P* < 0.05).” It is fully proved that the application of lymphocyte active immunotherapy for patients with unexplained recurrent miscarriage can predominantly optimize the estrogen indicators of patients.

In conclusion, prepregnancy lymphocyte active immunotherapy yields a promising therapeutic effect in the treatment of unexplained recurrent miscarriage. It not only ameliorates the patient's cellular immune function, but also further increases the patient's pregnancy success rate with a high safety, which is worthy of clinical application and promotion.

## Figures and Tables

**Table 1:** Comparison of clinical information between the two groups.

Categories	Experimental group (*n* = 62)	Control group (*n* = 62)	*χ* ^2^/*t*	*P*
Mean age (year)	27.34 ± 2.31	27.37 ± 2.34	0.072	0.943
Mean height (cm)	162.45 ± 3.42	162.51 ± 3.44	0.097	0.923
Mean pregnancy times	2.32 ± 0.34	2.34 ± 0.36	0.318	0.751
Education level				
University	31 (50.00%)	29 (46.77%)	0.129	0.719
Middle school	23 (37.10%)	24 (38.71%)	0.034	0.853
Primary school	8 (12.90%)	9 (14.52%)	0.068	0.794
Place of residence			0.134	0.714
Urban	24 (38.71%)	26 (41.94%)		
Rural	38 (61.29%)	36 (58.06%)		

**Table 2:** Comparison of pregnancy success rate between the two groups after treatment (*n* (%)).

Groups	*n*	Pregnancy success rate	Miscarriage rate
Experimental group	62	59 (95.16)	3 (4.84)
Control group	62	47 (75.81)	15 (24.19)
*χ* ^2^			9.359
*P*			0.002

**Table 3:** Comparison of estrogen indicators between the two groups of patients (*x̅*±*s*).

Groups	*n*	Estradiol		*β*-HCG		Progesterone	
		Before treatment	After treatment	Before treatment	After treatment	Before treatment	After treatment
Experimental group	62	62.45 ± 8.37	139.74 ± 20.48	14.26 ± 2.04	24.09 ± 4.37	22.13 ± 2.81	32.31 ± 5.53
Control group	62	62.54 ± 8.43	113.27 ± 46.74	14.37 ± 2.01	18.73 ± 3.21	22.26 ± 2.86	27.43 ± 4.41
*t*		0.060	4.084	0.302	7.784	0.255	5.433
*P*		0.953	≤0.01	0.763	≤0.01	0.799	≤0.01

**Table 4:** Comparison of hemorheology indexes between the two groups of patients (*x̅*±*s*).

Groups	*n*	HCT		EEP		ERP		PV	
		Before treatment	After treatment	Before treatment	After treatment	Before treatment	After treatment	Before treatment	After treatment
Experimental group	62	38.17 ± 5.13	25.66 ± 3.74	348.51 ± 22.11	293.12 ± 12.54	28.18 ± 6.23	15.19 ± 4.57	2.44 ± 0.57	1.23 ± 0.34
Control group	62	38.21 ± 5.07	32.43 ± 4.54	349.46 ± 21.87	325.18 ± 18.68	28.27 ± 6.34	21.73 ± 5.84	2.47 ± 0.61	1.72 ± 0.41
*t*		0.044	9.063	0.241	11.220	0.080	6.944	0.283	7.244
*P*		0.965	≤0.01	0.810	≤0.01	0.937	≤0.01	0.778	≤0.01

## Data Availability

The data used to support the findings of this study are available from the corresponding author upon request.
